# Improving visualization and analysis of relationships between neuronal model parameters in discrete parameter spaces

**DOI:** 10.1186/1471-2202-12-S1-P309

**Published:** 2011-07-18

**Authors:** Lisa McKee, Astrid A Prinz, Tomasz G Smolinski

**Affiliations:** 1 Department of Computer and Information Sciences, Delaware State University, Dover, DE 19901, USA; 2 Department of Biology, Emory University, Atlanta, GA 30322, USA

## 

We explore relationships between parameters in a 12-dimensional parameter space of a 2-compartment conductance-based model of the AB (anterior burster) neuron in the lobster stomatogastric ganglion (STG). The parameter space was created by systematically varying maximal conductances of membrane currents from a hand-tuned AB model [1] to determine ranges and variation steps that could potentially produce physiologically realistic behavior. This parameter space preparation contained between 3 to 5 discrete values for each of the 12 maximal conductances. Every parameter set representing an individual model neuron was simulated and classified as functional if it produced biologically realistic activity matching the behavior of the real AB neuron under several selection criteria [2]. Interactions between pairs of parameters in such a grid-based space can be visualized by 3D bar-plots, in which color, representing the third dimension, depicts the frequency of “good” models for each of the possible combinations of values for that pair. One can attempt to identify relationships between parameters (which, in turn, may point to a possible co-regulation of the corresponding ionic currents) by searching for “ridges” of high frequency (*i.e.*, “hot” colors) in such plots. However, due to the limited number of parameter values, identification of such interactions in the plot, as well as any numerical analysis to follow, can be difficult. In order to deal with this limitation, and at the same time to transform the data into more of a scatter-plot-like form (which is more suitable for numerical analyses such as regression modeling or curve-fitting), random jitter is often added to the discrete values to prevent mark overlapping [3]. Although this procedure may improve the visibility of relationships between parameters under some circumstances, in extreme situations, due to its randomness, it may actually obscure interactions otherwise present in the data. Therefore, we propose two methods for increasing the clarity and interpretability of such relationships in sparse parameter spaces. The overarching idea for these methods is that the information contained in the data can be used to amplify the visualization of the relationships in the corresponding displays. We extend the random jitter technique, by utilizing the “gravity effect” and the “jump effect,” so that areas of higher density affect the results of the jitter procedure. Rather than having the original parameter values vary purely randomly within the jitter limits, the procedures, while still random, incorporate a “bias” towards the areas of higher co-occurrence. In the gravity-based approach, for each combination of the parameter values, a set of vectors proportional to the frequencies of the neighboring combinations is calculated. Then, a resultant vector is computed to “pull” the jittered points along its direction. In the jump-based approach, the jittered data points “jump” towards one of the neighboring combinations, or stay within the original jitter area, based on a probability proportional to the frequency of each of the combinations involved. In both cases, if there is a relationship present in the data, it will be enhanced, making it easier to discern and analyze. On the other hand, if there is no relationship between a given pair of parameters, the plot will be equivalent to the regular jitter. Importantly, these techniques significantly augment our previous visualization approaches [4], by allowing identification of relationships that are non-linear. For instance, the relationship between the soma delayed-rectifier potassium, *I_Kd_*, and the transient calcium, *I_CaT_*, currents, which in our previous work was assumed to be linear, based on the current results, might actually have an exponential character.
